# Human Monoclonal Antibodies as Adjuvant Treatment of Chronic Hepatitis B Virus Infection

**DOI:** 10.3389/fimmu.2019.02290

**Published:** 2019-09-25

**Authors:** Antonella Cerino, Stefania Mantovani, Dalila Mele, Barbara Oliviero, Stefania Varchetta, Mario U. Mondelli

**Affiliations:** ^1^S.C. di Malattie Infettive II – Infettivologia e Immunologia, Dipartimento di Scienze Mediche e Malattie Infettive, Fondazione IRCCS Policlinico San Matteo, Pavia, Italy; ^2^Dipartimento di Medicina Interna e Terapia Medica, Università di Pavia, Pavia, Italy

**Keywords:** human monoclonal antibody, HBV—hepatitis B virus, B cells, immune system, adaptive immunity

## Abstract

Despite the availability of an effective prophylactic vaccine leading to sterilizing immunity, hepatitis B virus (HBV) is responsible for chronic liver disease in more than 250 million individuals, potentially leading to cirrhosis and hepatocellular carcinoma. Antiviral drugs able to completely suppress virus replication are indeed available but they are, by and large, unable to eradicate the virus. Several alternative new treatment approaches are currently being developed but none have so far captured the interest of clinicians for possible clinical development. A constant feature of chronic HBV infection is T-cell exhaustion resulting from persistent exposure to high antigen concentrations as shown by the high expression of programmed cell death protein 1 (PD-1) by HBV-specific CD8 T cells. One way of tackling this problem is to develop HBV-specific neutralizing antibodies that would clear excess envelope proteins from the circulation, allowing for nucleos(t)ide analogs or other antiviral drugs now in preclinical and early clinical development to take advantage of a reconstituted adaptive immunity. Several fully human monoclonal antibodies (mAb) have been developed from HBV-vaccinated and subjects convalescent from acute hepatitis B that show different properties and specificities. It is envisaged that such neutralizing mAb may be used as adjuvant treatment to reduce viral protein load, thus rescuing adaptive immunity in an effort to optimize the effect of antiviral drugs.

## Adaptive Immunity: The Main Player in HBV Control

Hepatitis B virus (HBV) is responsible for acute and chronic hepatitis, potentially leading to cirrhosis and hepatocellular carcinoma [reviewed in (1)]. Hepatitis B can be effectively prevented by a prophylactic vaccine (2), whereas, antiviral drugs are virtually unable to eradicate the virus in chronically infected individuals despite efficient suppression of HBV DNA replication (3). Adaptive immunity plays a major role to provide long-term control of infection; however, the very low frequency of circulating HBV-specific T cells in chronic infection contributes to the inability to clear the virus (4). Indeed, HBV may settle for life in occult form in the nuclei of hepatocytes as minichromosome (covalently closed circular DNA, cccDNA), despite apparent recovery, potentially reactivating in case of immune suppression (5). The persistence of cccDNA in hepatocytes is the main hurdle to eradicate HBV infection. The problem is further compounded by the rapid decline of T-cell and B-cell responses as a result of exhaustion induced by production of large amounts of excess HBV envelope proteins (6), largely resulting from integration of HBV DNA sequences into the host genome particularly in the HBeAg-negative chronic HBV infection [reviewed in (7)]. Of note, there is evidence that increased levels of HBsAg may contribute the CD8+ T cell dysfunction (8), and that HBsAg induces disruption of TLR9-mediated Interferon-alpha production by circulating plasmacytoid dendritic cells (9, 10). Moreover, HBV-specific T cells are mainly concentrated in the intrahepatic compartment together with a large number of HBV non-specific T cells (11), which may contribute to maintain liver inflammation via antigen-independent by-stander activation (12). Exhaustion caused by persistent exposure to high antigen concentrations provides the basis for T cell dysfunction, and results in up-regulation of programmed cell death protein 1 (PD-1) and other check-point molecules by HBV-specific CD8 T cells (13, 14). In line with this interpretation, the intensity of the T cell response appears inversely correlated with HBV DNA levels, with more intense HBV-specific responses detectable in patients with lower viral loads.

B-cell responses also play a fundamental role in HBV infection. Antibodies specific for HBsAg are critical for the neutralization of free extracellular HBV, thus preventing viral entry into susceptible hepatocytes (15). However, antibodies are unable to eradicate intracellular virus, a task fulfilled by MHC class I-restricted virus-specific CD8+ T cells by lytic and non-lytic mechanisms (16). Anti-HBs antibodies are also produced during the chronic phase of the infection in minimal amount, most likely complexed by the large amount of the HBV envelope proteins present in the serum. Antibodies to the viral nucleoprotein, anti-HBc and anti-HBe, instead persist during chronic infection and detection of anti-HBe is linked to the emergence of the e-minus variant. The finding of undetectable HBV DNA by standard assays and anti-HBc in the absence of HBsAg is taken as surrogate evidence of occult HBV infection (17).

Current evidence supports the view that a coordinated activation of both T and B cells is critical for eradication of infection. Of note, CD4+ T cells control CD8+ T cell activity and antibody production (18), and the inability of the infected host to mount robust virus-specific antibody responses correlates with a premature loss of CD4+ T cell responsiveness during the establishment of viral persistence in a chimpanzee model of HBV infection (19, 20). Interestingly, sera containing high-titer anti-HBs antibodies provide protection upon exposure to HBV (21), the eradication of which is closely related to the development of neutralizing anti-HBs antibodies. The discovery of the sodium taurocholate cotransporting polypeptide (NTCP) as the receptor used by HBV to bind and infect liver cells allowed identification of the preS1 sequence which is used by HBV to bind liver cells and to characterize the role of anti-preS1 antibodies in the inhibition of HBV entry and infectivity (22). The potency and efficiency of anti-hepatitis B surface antibodies in controlling HBV also comes from early evidence showing that hepatitis B vaccines may be efficacious even when given after exposure (2) and that administration of anti-HBs immunoglobulin can prevent reinfection of liver grafts (23).

## The Neglected Role of B-cell Responses in Chronic HBV Infection

Humoral immunity seems to play an important, but only partially understood role in the control of HBV infection (24). This is also exemplified by the observation that treatment of individuals with manifest or occult chronic HBV infection with specific anti-B cell therapy, e.g., the anti-CD20 monoclonal antibody rituximab, to treat autoimmune disease or non-Hodgkin lymphomas, may carry a substantial risk of HBV reactivation, even those who achieved HBsAg clearance and seroconversion to anti-HBs. Indeed, the rate of reactivation after rituximab is higher than for similarly matched patients treated with T cell immunosuppressive agents (25, 26), pointing to the importance of B cell responses in the control of HBV.

In contrast with the wealth of studies on antibody responses to HBV proteins, there is limited information on the B-cell phenotypic and functional features during HBV infection, particularly during the chronic phase. Of note, and in contrast with evidence of exhausted T cells in the peripheral blood of patients with chronic HBV infection, bulk peripheral blood B cells express several activation markers and produce large amounts of immunoglobulin in response to innate and adaptive immunity signals, significantly more than B cells from patients with chronic HCV infection (27). In agreement with this vigorous polyclonal B-cell activation there was no evidence of upregulation of B-cell exhaustion markers, i.e., FcRL4, adding further support to the concept that the bulk B cell population apparently remains functionally intact in this setting. This would explain why in common practice patients with chronic HBV infection maintain the ability to produce antibodies to recall antigens and are able to respond to soluble protein vaccines. However, recent evidence indicates that, after enrichment, HBsAg-specific B cells show a phenotype of atypical memory B cells, a functionally defective subset which lack expression of CD21 and CD27 resulting from chronic pathogen exposure, particularly HIV infection (28). These B cells demonstrated altered signaling, homing, differentiation into antibody-producing cells, survival and antiviral/proinflammatory cytokine production, that could be partially rescued by PD-1 blockade (29). These findings suggest that persistent HBV infection drives an accumulation of atypical antigen-specific B cells with impaired antiviral capacity. These findings were akin to a simultaneously published study (30), which suggested that B cell alterations were not limited to HBsAg-specific B cells but affected the global B cell population. However, this last finding would apparently be difficult to reconcile with previous evidence of conserved global B-cell function in chronic HBV infection (27). Recent, interesting work from one of these two highly reputed laboratories shed light on the matter. Indeed, a comparative characterization of B cells specific for the HBV nucleocapsid and envelope proteins showed that HBcAg-specific B cells prevailed over HBsAg-specific in patients with chronic HBV infection and were able to efficiently differentiate into immunoglobulin-secreting cells, compared with HBsAg-specific B cells. Moreover, HBcAg-specific B cells have a classical phenotypic and transcriptome profile of IgG+ memory B cells, whereas HBsAg-specific B cells were confirmed to have an atypical memory B cell profile. This is one of the first reports of dichotomous B-cell differentiation according to antigenic specificity in viral infections and provides a plausible explanation for the higher anti-HBc titers, relative to low anti-HBs titers observed in sera from patients with chronic hepatitis B (31).

A recently identified family of regulatory cells, regulatory B cells (Bregs), was reported to control immune responses at the innate and the adaptive levels, and only a few studies have investigated the role of Bregs in chronic hepatitis B. Bregs classically suppress immune function through secretion of the inhibitory cytokine IL-10, which inhibits the production of pro-inflammatory cytokines and supports regulatory T cell differentiation. One study reported increased Breg cells in PBMC of patients with chronic HBV infection during spontaneous hepatic flares, demonstrating suppressive IL-10 mediated activity of Bregs on HBV-specific CD8 cells *in vitro* (32). Enhanced serum levels of IL-10 have indeed been described in chronic hepatitis B, particularly in the so-called immune activation phase (33). However, such temporal association with necro-inflammatory flares may either suggest a direct Breg responsibility via suppression of HBV-specific CD8 T cells, therefore diminishing viral control, or a secondary Breg-mediated modulation of the inflammatory flare induced by a virus-specific CD8 T cell response, to switch-off the exaggerated immune activation. Both scenarios probably apply to most chronic infections but further data are needed to better understand the role of Breg cells in this clinical setting.

## Human Monoclonal Antibodies to Treat HBV Infection?

Human memory B cells and plasma cells are a major source of antibodies. Indeed, B cells undergo somatic mutations and selection by antigen and T-cell help in response to pathogens and persist for life as memory cells in a given individual, rapidly responding to booster immunization to yield a large number of plasma cells. Even though long-lived plasma cells are the main source of serum antibodies, these terminally differentiated cells are certainly not ideal for long-term culture. Several methods have been developed to isolate human monoclonal antibodies (humAbs). The original relatively simple approach consists of the isolation of single antigen-specific Epstein-Barr virus (EBV)-immortalized B cells from donors with high-titer specific immunoglobulin (Ig) after labor-intensive, time-consuming rounds of cloning steps (34). This approach is limited by the instability of the cell lines, the low level of specific antibody secretion, and the poor cloning efficiency, yielding low numbers of Ag-specific clones, usually <0.5% of the initially seeded wells. Improvements of the original methodology are represented by enrichment of antigen-specific B cells using fluorochrome-conjugated antigen to capture B cells of interest, thus increasing the chances of obtaining specific antigen-specific B-cell clones [reviewed in (35)]. This method has been successfully used, but potential technical issues pertaining to the antigen used may represent an unsurmountable hurdle. Another technique that has been extensively used includes phage-displayed antibody libraries with random pairs of antibody heavy and light chains, enabling the use of appropriate target antigens to screen specific antibodies with high affinity to the antigen (35). Humanized transgenic mice may also provide an alternative option to obtain fully human therapeutic antibodies. In humanized transgenic mice, the endogenous murine antibody gene is replaced by human Ig loci (36). Transgenic mice are then immunized with Ag to elicit specific human antibodies. Alternative approaches to improve the cloning efficiency of the original methodology have been considered, including selection of memory (CD27+) B cells and EBV immortalization and delivering an innate immunity signal, with a TLR9 agonist, such as CpG oligodeoxynucleotides, for potent activation of memory B cells (37). EBV-transformed B-cells can then be further stabilized by fusion with non-Ig secreting mouse-human myeloma heterohybrids (34). Using this approach, we have generated a number of humAbs specific for hepatitis C virus (HCV) (38–40) and more recently for HBV (41). Among the HBV-specific humAbs developed, one derived from a vaccinated individual showed extremely potent neutralizing activity in a *Tupaia belangeri* model of HBV infection *in vivo*, whereas others apparently did not or had a low neutralization titer (41). Interestingly, the humAb showing the highest neutralizing activity recognized a conformational epitope within the common “a” determinant in the major envelope protein (S), whereas, the remainder obtained from acute hepatitis B convalescents recognized linear epitope(s) in the same envelope polypeptide (41). The data were not entirely superimposable to those obtained with a NTCP-expressing HepG2 cell line, as some of the *in vivo* poorly neutralizing humAbs were, instead, efficiently neutralizing in the NTCP-expressing cell line *in vitro* system (unpublished data). The anti-HBV humAbs were originally developed to obtain sustainable reagents for immunoprophylaxis of HBV re-infection in liver transplant recipients and other settings, such as treatment of accidental needle-sticks and in the prevention of vertical-perinatal HBV transmission. Anti-HBs antibodies are usually administered as an additional prophylactic measure while waiting for the appearance of neutralizing anti-HBs antibodies. However, it has not escaped our attention that another potentially important application may emerge in the future as an adjuvant treatment of chronic HBV infection.

## Perspective For a Combined Use of Humabs and Nucleos(t)ide Analogs for HBV Treatment

**Figure 1:**
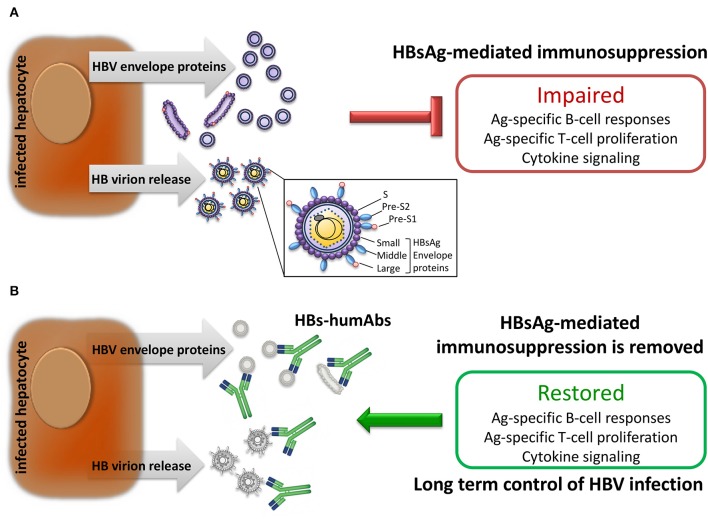
Effect of HBV infection on immune responses. **(A)** Infected hepatocytes produce and release large amounts of HBV envelope polypeptides also expressed on HB virions which suppress adaptive immune responses. **(B)** Human monoclonal antibodies specific for HBV envelope proteins (HBs-humAbs) may reduce peripheral protein load allowing restoration of B and T cell-mediated immune responses.

**Figure 2:**
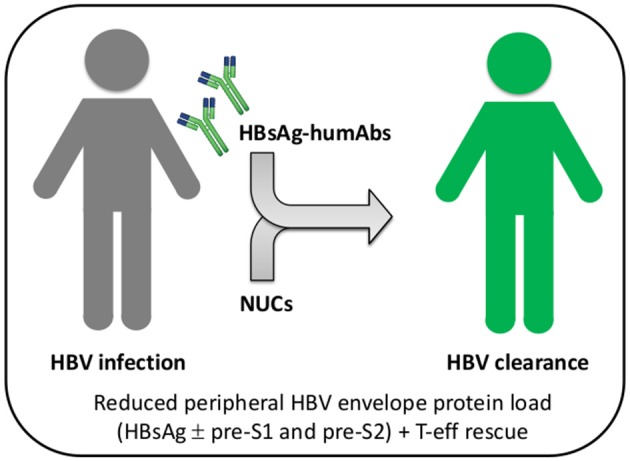
Combined or sequential treatment of chronic HBV infection with HBsAg-specific humAbs and nucleos(t)ide analogs. Patients with chronic HBV infection (gray) can be treated with HBs-humAbs to rescue T and B cell exhaustion caused by HBV envelope proteins, either sequentially or in combination with nucleos(t)ide analogs (NUCs). Reconstitution of HBV-specific adaptive immunity would ultimately increase NUC efficacy and result in viral clearance (green). T-eff, T effector cells.

Antibodies are usually effective in prophylaxis if given shortly after viral exposure, their efficiency declining after infection. However, highly potent and/or broadly cross-reactive human monoclonal antibodies may counteract viral escape mechanisms and open new avenues for intervention. For instance, potent HIV-1 envelope-specific broadly neutralizing antibodies (super-antibodies) have been identified that may have potential therapeutic applications (42). In chronic hepatitis B, the potential advantage of anti-HBs humAb therapy relies on the possibility to regenerate effective T cell responses in patients with chronic HBV infection whose T cells are dysfunctional as a result of exposure to very large amounts of HBV envelope proteins produced in excess during viral replication. Indeed, removal of circulating HBsAg by neutralizing anti-HBs mAb was reported to rescue the exhausted adaptive immune responses to hepatitis B vaccination, eventually leading to anti-HBs seroconversion in a murine model (43). It may, thus, be possible to apply this concept to treatment of chronic hepatitis B (Figure 1). Notably, a murine anti-HBs mAb was employed to treat hypogammaglobulinemic HBV-infected individuals yielding reduced HBV DNA levels (44). Moreover, a human anti-HBs mAb, alone or in combination with interferon-α, was used in patients with chronic HBV infection, resulting in a substantial decrease in HBsAg titers (45). Interestingly, combination therapy with interferon-α successfully cleared HBsAg. Therefore, combined use with HBV nucleos(t)ide analogs can be definitely envisaged (Figure 2). It is important to emphasize that a potentially dangerous effect of immune complex formation following intravenous injection of monoclonal or polyclonal anti-HBs antibodies in HBsAg positive mice, chimpanzees and human subjects has so far not been convincingly demonstrated. Indeed, no renal complications or immune complex disease has been described in this setting. Similarly, no untoward effects were reported in HBV-infected individuals receiving high doses of polyclonal hepatitis B immunoglobulin (46). It is anyway important, in principle, that anti-HBs humAbs be administered in molar excess to ensure the presence of excess uncomplexed humAbs for effective neutralization and elimination of circulating HBV.

Cooperation between the humoral and cellular arms of adaptive immunity is required for efficient elimination of pathogens. To this end, antiviral humAbs can be exploited to mediate antibody-dependent cell-mediated cytotoxicity (ADCC), which takes place via specific receptors recognizing the Fc fragment of IgGs (FcγRs) expressed on the membrane of several cells involved in innate and adaptive immune responses. The FcγR family includes several classes of receptors that show different affinity for the Fc fragment. One of these, the low affinity FcγRIII (CD16), is the most potent activating receptor of NK cells mediating ADCC (47). To evaluate the potential to mediate ADCC, an assay has been developed to measure the ability of antiviral IgG triggering activation of cells expressing Fcγ receptors (48). This is obtained by the interaction of chimeric Fcγ receptors with immune complexes formed on the surface of IgG-coated target cells resulting in FcγR-mediated activation, which makes use of IL-2 secretion by a mouse hybridoma as a surrogate readout. It is thus possible to screen different humAbs for their potential to efficiently mediate ADCC. Whether ADCC plays a role in the setting of HBV infection is presently unclear, but would also depend on the ability of NK cells and other innate lymphoid cells to perform this function, as well as on the affinity of FcγRIII (CD16). Of note, studies using a mAb targeting a linear epitope (aa 119–125 of HBsAg on the first loop N-terminus located at the major hydrophilic region of HBsAg) promoted HBV clearance and blockade of viral infection (49). Even more interestingly, other humAbs targeting the NTCP-binding site of preS1 (50) were able to reduce HBV DNA and HBsAg levels in murine models of established HBV infection and efficiently mediated ADCC, which was abolished when these antibodies were engineered in the Fc to prevent binding to FcγRs.

The findings discussed above suggest that humAbs targeting HBV envelope epitopes might indeed have a potential for combination therapies in chronically infected individuals. Phase I clinical trials are therefore eagerly awaited in this setting.

## Author Contributions

MM wrote and conceived the manuscript. AC, DM, and SM contributed to conception and generated data. BO, SV, and SM revised the manuscript, read, and approved the submission.

### Conflict of Interest

The authors declare that the research was conducted in the absence of any commercial or financial relationships that could be construed as a potential conflict of interest.
